# Interplay of Forces and the Immune Response for Functional Tendon Regeneration

**DOI:** 10.3389/fcell.2021.657621

**Published:** 2021-06-04

**Authors:** Yuwei Yang, Yicong Wu, Ke Zhou, Dongmei Wu, Xudong Yao, Boon Chin Heng, Jing Zhou, Hua Liu, Hongwei Ouyang

**Affiliations:** ^1^Dr. Li Dak Sum & Yip Yio Chin Center for Stem Cells and Regenerative Medicine, and Department of Orthopedic Surgery of The Second Affiliated Hospital, Zhejiang University School of Medicine, Hangzhou, China; ^2^Zhejiang University-University of Edinburgh Institute, Zhejiang University School of Medicine, and Key Laboratory of Tissue Engineering and Regenerative Medicine of Zhejiang Province, Zhejiang University School of Medicine, Hangzhou, China; ^3^Central Laboratories, School of Stomatology, Peking University, Beijing, China; ^4^Department of Sports Medicine, Zhejiang University School of Medicine, Hangzhou, China; ^5^China Orthopedic Regenerative Medicine Group (CORMed), Hangzhou, China

**Keywords:** tendon regeneration, biomechanical properties, immune response, stem cells, forces

## Abstract

Tendon injury commonly occurs during sports activity, which may cause interruption or rapid decline in athletic career. Tensile strength, as one aspect of tendon biomechanical properties, is the main parameter of tendon function. Tendon injury will induce an immune response and cause the loss of tensile strength. Regulation of mechanical forces during tendon healing also changes immune response to improve regeneration. Here, the effects of internal/external forces and immune response on tendon regeneration are reviewed. The interaction between immune response and internal/external forces during tendon regeneration is critically examined and compared, in relation to other tissues. In conclusion, it is essential to maintain a fine balance between internal/external forces and immune response, to optimize tendon functional regeneration.

## Introduction

Tendon is a key component of the musculoskeletal system, which physically connects muscle with bone and transmits mechanical forces from muscle to bone. The significant role of tendon tissue in human locomotion is determined by its function and position. However, tendon is easily injured due to unconscious overuse and has limited healing and regenerative capacity. This is best illustrated by a study related to the National Basketball Association, whereby more than a third of players ended their careers prematurely, or were unable to regain their top performance after Achilles tendon rupture, due to deterioration of tendon mechanical properties (Lemme et al., 2019). It was also shown that 38.4% of professional football players suffered subsequent re-injury after their first Achilles tendon injury (Ekstrand et al., 2020). Clinically, biomechanical properties, particularly tendon tensile strength, are often related to the extent of tendon repair (Frankewycz et al., 2018; Feichtinger et al., 2019). It is well-known that tendon injury always induces an immune response, which may further lead to further loss of tensile strength, making the repaired tendon susceptible to repeated injuries. Inflammation is an important driver of tendon healing, but persistent inflammation causes tendon fibrosis and other matrix changes (Dakin et al., 2014). Therefore, delineating the relationship between immune response and tensile strength is crucial for optimizing tendon regeneration. Besides, external tensions including physiotherapy (Evans and Thompson, 1993) and biomaterials (Sawadkar et al., 2020) providing mechanical support can regulate inflammation. In this review, we will critically examine the effects of internal/external forces and immune responses, particularly the interplay of internal/external forces and the immune response on tendon regeneration.

## Contribution of Normal Tendon Structural Basis to Tendon Biomechanics

The key of tendon biomechanics is tensile strength (Wu et al., 2018). Tensile strength reflects the maximum tensile load that tendon is capable of enduring, and it is closely related to elastic modulus, which is an important index to evaluate tendon repair effect. In clinical studies, the elastic modulus of human Achilles tendon is over 300 kPa, which will decrease to 3–200 kPa after Achilles tendon rupture (Chen et al., 2013). For tendons at other sites, the average tensile strength of tendons with tendinitis was significantly reduced by three times compared with healthy tendons (Dirrichs et al., 2016). In the locomotor system, the average elastic modulus of healthy muscle, which also belongs to soft tissue, is only 20 kPa (Nordez and Hug, 2010), which is 15-fold lower than that of tendon (Feng et al., 2018). The average Young’s modulus of skin is between 4.5 and 8 kPa (Pailler-Mattei et al., 2008), which is far less than the mean value of tendon. In contrast, compared with the hard tissue, the average modulus of elasticity of cartilage is 500 kPa (Wilusz et al., 2013), which is higher than that of tendon. This is mainly related to the different ways to undergo mechanical stress in soft and hard tissue; that is, the soft tissues mainly undergo mechanical tension, and the hard tissues mainly undergo mechanical compression. Hence, in the case of the same way to undergo mechanical stress, the tensile strength of tendon is much higher than that of other soft tissues. Those are why tensile strength is considered the key characteristic biomechanical parameter of tendon. The excellent tensile strength of tendon depends on the normal tendon structural basis.

**FIGURE 1:**
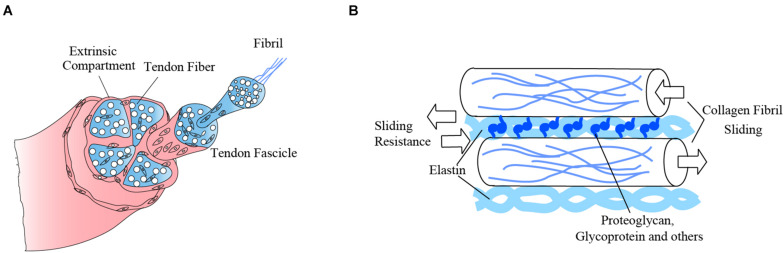
The biomechanics of different components of tendon matrix ensure tensile strength of the whole tendon. **(A)** The main component of tendon is collagen. The base of tendon tissue is collagen fibers, which consist of collagen fibrils. **(B)** The tendon matrix components, collagen fibrils, elastin fibrils, proteoglycan, glycoprotein, and other macromolecules, combine with each other to provide micromechanics. These micromechanics interact and give rise to the macro-mechanics of tendon, which enable its biomechanical function.

Tendon is a type of sparsely vascularized soft tissue (Lehner et al., 2016), with abundant extracellular matrix (ECM), but sparsely populated with a few cell types (Figure 1). Tendon ECM contains collagen, elastin, proteoglycan, glycoprotein, and other macromolecules (Thorpe and Screen, 2016). Collagen is the major component of tendon, which accounts for 60–85% of tendon dry weight (Monti and Miyagi, 2015). On day 14 of chicken embryo development, tendon structure begins to be established by pre-fibrils (Birk et al., 1989). In this stage, tendon shows a weak capability to transmit force, which means the beginning of limb movement (McBride et al., 1988). Collagen fibrils are aggregated into collagen fibers after day 17 or 18 of chicken embryo development (Birk and Zycband, 1994). In this stage, it shows a mechanical characteristic of mature tendon (McBride et al., 1988). The collagen fibers are the basis of the excellent biomechanical properties of tendon by functioning as tensile-resistant fibers (Screen et al., 2015). By spreading the tension throughout the entire tendon rather than just a small part of the tissue, tensile strength is enhanced (Kannus, 2000).

Elastin is one type of non-collagenous matrix molecule within tendon that plays an essential role in tendon biomechanical properties. In tendon, elastin forms a three-dimensional crisscross network and links adjacent collagen fascicles. The elastin network appears in developing tendons in the embryo between days 7.5 and 8 (Hurle et al., 1994). It can help collagen fibers transfer mechanical forces and protect them from excessive shear (Hill et al., 2020) through close physical linkage with adjacent collagen fibrils. Additionally, the elastin network envelops and protects cells (Pang et al., 2017). Besides elastin, various proteoglycans, glycoproteins, and other molecules also play important roles in regulating collagen fibrillogenesis. These provide non-covalent interactions by being integrated within the collagen fibrils (Birk et al., 1989) and help resist collagen fibril sliding, which enhances tendon viscoelasticity and decreases failure loads (Redaelli et al., 2003; Robinson et al., 2017), thus ensuring good tendon biomechanical properties. Taken together, the orderly and complex structure of tendon ECM is the basis of the excellent biomechanical properties of tendon, and this relationship has been established during embryonic development.

There are two main cell types in tendon, tenocytes, and tendon stem cells. Tenocytes are active in synthesizing and secreting collagen (Kannus, 2000). The tenocyte gap junctions are also considered as essential mediators of mechanosensitive responses (Maeda et al., 2012; Wu et al., 2018) that regulate tendon ECM synthesis and degradation (Young et al., 2009). Tendon stem cells are known as tendon stem/progenitor cells (TSPCs) or tendon-derived stem cell (TDSCs), which are functionally similar to mesenchymal stem cells (MSCs) (Docheva et al., 2015). TSPCs possess the capacities of self-renewal, clonogenicity (Bi et al., 2007), and differentiation (Magne and Bougault, 2015). These cells also contribute to biomechanical properties of embryonic tendon by their actin cytoskeleton network. Between days 8 and 11, actin cytoskeleton network related to high cell density and contact shows a similar spatial structure as collagen fibers, which is considered to provide tendon with biomechanical force (Schiele et al., 2015). The actin cytoskeleton network of cells in tendon can provide more than 20% elastic modulus (Schiele et al., 2015). In contrast, the contribution of myocytes to the elastic modulus of muscle tissue is 13–21% (Collinsworth et al., 2002), and chondrocytes, together with their ECM, provides about 12% of Young’s modulus of cartilage (Alexopoulos et al., 2003). These findings suggest that although the tendon is mainly composed of ECM, the cells in tendon also affect tendon mechanics to a certain extent. Compared with other tissues in the locomotor system, tendon cells have a greater impact on tissue mechanics, which also suggests that we need to pay more attention to the metabolic activities of cells in the tendon in the process of tendon functional regeneration.

In adult injured tendon, histological results revealed that collagen are often in a disorganized state. The population of tenocytes is increased compared with normal tendon (Li and Hua, 2016) and TSPCs trended toward chondrogenic and osteogenic differentiation (Docheva et al., 2015). For this reason, more effective therapies should be developed in the future to enable tendon to achieve better regenerative effects, as close to the normal state as possible. At the cellular level, the ultimate goal of tendon regeneration is the normalization of cell number and ECM structure, as a basis for functional recovery of injured tendons.

## Inflammation in the Process of Tendon Diseases Regulates Tendon Biomechanics

The deterioration of tensile strength is mostly due to disordered tendon matrix caused by a failed healing response in tendon diseases (Maffulli et al., 2010). There are three different pathogenesis of tendon diseases—genetic diseases, tendinopathy, and tendon rupture (Gaut and Duprez, 2016). Tendinopathy and tendon rupture are caused by external factors. Tendinopathy is a typical tendon disease, mostly manifesting after minor injury under long-term repeated overstress of tendon collagen fibers, which leads to chronic inflammation (Longo et al., 2009). In this process, inflammation does not subside and apoptotic cells fail to be cleared. With the stimulation of inflammation, tenocytes show high expression of stromal fibroblast activation markers to develop fibrosis (Dakin et al., 2018). Tendon rupture results from instantaneous overloading of external force or clinical operation. Compared with chronic inflammation, inflammation caused by rupture is more acute (Klatte-Schulz et al., 2018). Both tendinopathy and rupture can induce heterotopic ossification in tendon because of inflammation (Łęgosz et al., 2018). It has also been shown that tendon heterotopic ossification developing after rupture or tendinopathy will further decrease tensile strength, with calcium deposition in the tendon matrix and disordered collagen arrangement (Zhang et al., 2016). Furthermore, it has been demonstrated that, without the participation of the immune response, structural changes in tendon matrix induced by other environmental cues such as high glucose (Wu et al., 2017) contributes to the loss of tensile strength in tendon (Guney et al., 2015).

During inflammation, fibroblasts are recruited by immune cells to alter collagen arrangement, leading to negative changes in tensile strength (Best et al., 2019). At some time, suppression of macrophages can also cause increase of tensile strength because of high TGF-β3/TGF-β1 ratio similar to that in fetuses (De La Durantaye et al., 2014). This means that inflammation is actually one of the key factors that influence tendon functional regeneration by improving tensile strength. Furthermore, mechanosensitive proteins are usually influenced by the immune response, taking part in tendon regeneration. Take, for example, cystic fibrosis transmembrane conductance regulator (CFTR), one type of tension-mediated activation channel, which has been identified to be mechanosensitive under regenerative conditions in tendon (Liu et al., 2017). A further study confirmed that the anti-inflammatory molecule annexin A1 is a promising target of CFTR (Liu et al., 2018). This means that immune response takes part in the healing pathway involving some mechanosensitive genes or proteins that promote tendon regeneration.

## Immune Response Drives Tendon Repair

Inflammation is the main inducer of biomechanical decline in the process of tendon diseases; however, it is also one part of immune response that drives tendon repair. The tendon repair process can be extrinsic or intrinsic (Ingraham et al., 2003). The intrinsic repair process usually occurs during the fetal period in mammals (al-Qattan et al., 1993) or non-mammalian vertebrates (Andarawis-Puri et al., 2015) with minimal inflammatory cell infiltration and fibrosis (Ehrlich et al., 2005; Menzies et al., 2016). The extrinsic repair occurs in adult tendon, accompanied by inflammation (Koob and Summers, 2002). During intrinsic repair, the injury site has low ratio of TGF-β1/TGF-β3, which is opposite to extrinsic repair (Ferguson and O’Kane, 2004). During extrinsic repair, excessive inflammatory response causes disruption of tendon ECM homeostasis, resulting in fibrosis and adhesive scar formation (Wu et al., 2019). Fibrosis and adhesive scar formation cause loss of biomechanical properties, which make these an extremely serious problem. During this process, fibroblasts proliferate and migrate from the epitenon and endotenon into the injured area, together with immune cell infiltration.

**FIGURE 2:**
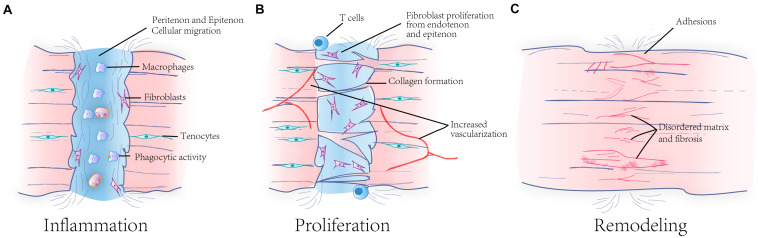
Three phases of tendon healing and the matrix changes during the healing process. Tendon healing goes through three phases, including inflammation **(A)**, proliferation **(B)**, and remodeling **(C)**. In the inflammation phase, immune cells and fibroblasts from peritenon and epitenon migrate into injured position. In the proliferation phase, fibroblasts proliferate and angiogenesis occurs in this time. In the remodeling phase, repaired tendon has disordered matrix. The injured tendon finally forms tendon fibrosis and adhesion.

In general, with extrinsic repair of tendon, there are three stages involving wound healing, inflammation, proliferation, and rebuilding (Hope and Saxby, 2007; Figure 2). The changes in cell population and morphology and the compositions of matrices and molecules in peritendinous and intratendinous tissues are taken as the boundary standard of these three stages (Docheva et al., 2015). Firstly, the inflammatory stage is the most responsive stage after tendon injury. In this stage, there are significant inflammatory infiltrations of mast cells (Alim et al., 2017) and macrophages (Millar et al., 2010), with pro-inflammatory cytokines being present (Millar et al., 2008), as a result of platelet aggregation. Macrophages are the primary cells involved during the inflammatory stage, leading the direction of tendon healing. They release cytokines to trigger further inflammation (Hudgens et al., 2016). Mast cells are related to angiogenesis (Jetten et al., 2014) and pro-inflammatory response to tendon cells (Chisari et al., 2020), which can not only promote wound healing but also lead to decrease of the synthesis and degradation (Behzad et al., 2013) of tendon matrix. In this process, tendon cells will also respond to the inflammatory environment, regulate the production of type I collagen, and affect macrophage polarization through intercellular communication (Stolk et al., 2017). During the second stage, stromal fibroblasts are recruited and proliferate, in response to some cytokines such as interleukin (IL)-1β (Dakin et al., 2018) produced by inflammatory cells. At the same time, angiogenesis can also be observed (Oshiro et al., 2003). It is worth mentioning that in the injured tendon, the ratio of type I to type III collagen is lower than the native tendon (Williams et al., 1980). The reason is that the production of type III collagen increases because of the rapid proliferation of stromal fibroblasts. The rebuilding stage begins at this time point. At this stage, the synthesis of type I collagen is predominant, instead of type III collagen, and the organized collagen fibrils is progressively arranged into bundles. However, the ratio will not return to the normal level as before. Finally, fibrosis and excessive angiogenesis will result in further development into tendonitis. Apart from that, a few studies have reported that adaptive immune responses also exist after tendon injury. In the early inflammatory response, macrophages and dendritic cells present antigens, and dendritic cells allow rapid accumulation of T cells and B cells. Accordingly, a peak in the number of dendritic cells and CD4^+^ T cells can be observed after 2 weeks of injury, while the number of B cells and CD8^+^ T cells increases with time (Blomgran et al., 2016; Noah et al., 2020). During this process, CD4^+^ T lymphocytes lead to an increase in fibronectin around tendon, providing a scaffold for the subsequent formation of adhesions, thereby reducing the mechanical properties of tendons (Wojciak and Crossan, 1993). It has been shown that the use of corticosteroids during the adaptive immune response of tendon healing reduces the number of CD8a^+^ cytotoxic T cells, improving tendon healing (Blomgran et al., 2017). Although studies of innate and adaptive immune responses during tendon healing has been a lot, there is still much that remains to be studied, especially the specific effects of adaptive immunity on the tendon matrix structure.

Nevertheless, the immune response is more about playing a positive role in promoting tendon regeneration, particularly with the involvement of activated macrophages (Millar et al., 2017) and related cytokines (Gelberman et al., 2017). There are two main types of macrophages, M1 and M2. M1 macrophages exert a pro-inflammatory role during the early repair phase, yet M2 macrophages are able to exert anti-inflammatory effects to improve regeneration (Mauro et al., 2016). As a result, compared with M1 macrophages, more attention is paid to M2 macrophages, due to their more significant role in tendon regeneration. Recently, M2 macrophages are found to be able to accelerate regenerative responses in tendon healing (Chamberlain et al., 2019). Several recent therapeutic approaches targeting macrophages have also shown that increasing the proportion of M2-type macrophages during tendon healing improves tendon biomechanical performance (Sunwoo et al., 2020). Moreover, inflammatory factors and cytokines are considered to indirectly regulate immune cells like macrophages during tendon scarless healing (Gelberman et al., 2017; Best et al., 2019). For adaptive immunity, a specialized T cell population is also present in tendons, which shows CD4 and CD8 double-negative. This population secretes IL-22 and potentially mediates tendon-to-bone healing (Sherlock et al., 2012; Abraham et al., 2017).

Furthermore, biomaterials can be applied to regulate the inflammatory response to promote tendon healing. In this regard, the main consideration is given to the bacteriostatic effect, biocompatibility, and modulation of immune cells of biomaterials. The bacteriostatic effect and biocompatibility of biomaterials are the most intensively considered aspects in tissue engineering. A study of biomaterials commonly used in tissue engineering has shown that hyaluronic acid has a significantly higher bacteriostatic effect than type I collagen and PLGA. Type I collagen has better biocompatibility in tendons, but *Staphylococcus aureus* can still establish itself in collagen scaffolds with high concentration. However, PLGA is less biocompatible and generates an acidic environment at the implantation site, which induces inflammatory reactions (Carlson et al., 2004). In addition, the new silk scaffolds used for tendon repair can enhance the maturation of dendritic cells and induce the generation of early immunity (Musson et al., 2015). Therefore, the selection of an appropriate biomaterial is very important for tendon repair. The modulation of immune cells by biomaterials has been extensively studied. The effects of biomaterials on macrophage polarization in tendon repair have been summarized; that is, biomaterials with ECM coatings, hydrophilic surfaces, and nanometer sizes can induce macrophage activation (Lin et al., 2018), whereas in recent years, other properties of biomaterials have also been shown to regulate macrophages. Magnetic materials, for example, have been shown to induce macrophage to M2 transition (Vinhas et al., 2020). The fiber arrangement of biomaterials also influences the polarization of macrophages to promote tendon repair (Schoenenberger et al., 2020).

## Interplay of the Immune Response and Forces Improves Tendon Regeneration

In the regeneration of other tissues, such as cartilage and bone, the relationship between forces and immune response after injury has been characterized (Guilak et al., 2008; Raghunathan et al., 2017) and their interaction influences the regenerative effect. It has been demonstrated that external stimulation of cartilage are associated with inflammation and exert a profound influence on cartilage regeneration (Chen M. et al., 2018). Physical stimulation also causes sensitive response of pro-inflammatory mediators around injury areas (Fahy et al., 2019). These findings directly validate the potential influence of external forces on inflammation. Inflammation also facilitates the recovery of mechanical properties as well. One type of transcriptional regulator induced by immune cells is a mediator of the crosstalk between IL-4 and mechanical-induced signaling pathways, which suppresses the degradation of cartilage matrix (He et al., 2019). As we can see from this study, the interplay of immune response and forces improves cartilage healing. In view of its good prospect for clinical applications in other tissue regeneration therapies, it is worthwhile considering studying it in tendon.

**FIGURE 3:**
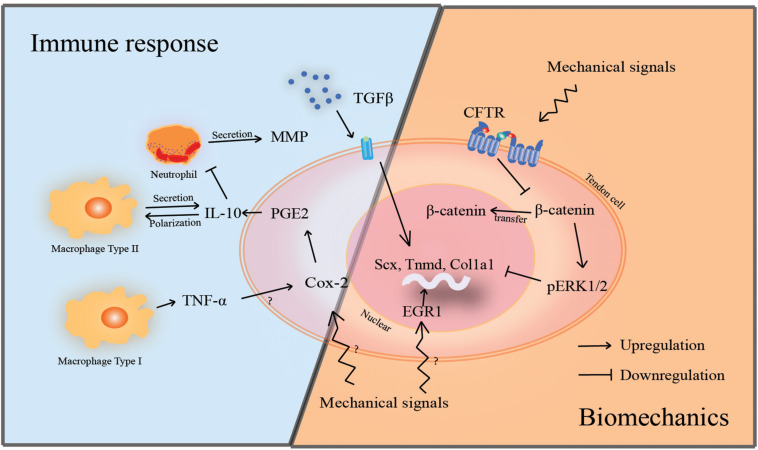
Both mechanical signals and immune response can regulate tendon cells and further influence tendon regeneration. Inflammation and mechanical signals can control the tenogenic differentiation by regulating the transcription of tendon markers in tendon cells. Some cytokines and mechanical signals have the same targets, indicating a kind of potential co-regulation pathway. However, most mechanosensitive proteins are unknown, and the inflammation process still needs a deeper understanding. In that, it is necessary to figure out the mechanism of the balance between biomechanics and immune response during tendon regeneration.

The tune-up of the immune response and forces in tendon could be described in two parts (Figure 3). Firstly, the influence of immune response on tensile strength, and secondly, the influence of external forces on immune response. On one hand, immune response influences tensile strength, as mentioned above. On the other hand, cyclic stretching or overloading tends to trigger strong immune responses that will exacerbate tendinopathy (Chen Q. et al., 2018; Schoenenberger et al., 2018). Specifically, microinjuries of collagen fibrils resulting from overloaded stretch that eventually deform to 10–15% (Chen Q. et al., 2018) can also significantly reduce cell viability within tendons, while microinjuries that accumulate within tendon collagen fibrils ultimately activate immune responses (Stauber et al., 2020), increase expression of inflammatory markers, and lead to degradation of the tendon matrix (Thorpe et al., 2015). Cyclic stretching on tendon fibroblasts also produces a significant elevation in the expression of the inflammatory-related factor leukotriene B4 (Li et al., 2004). In an *in vivo* experiment, cyclic loading at low strength also caused an increase in the number of inflammatory macrophages at the tendon-to-bone healing after anterior cruciate ligament (ACL) reconstruction (Brophy et al., 2011). This implies the influence of mechanical loading on tendon-to-bone healing after clinical ACL reconstruction surgery. It also supported the idea that the use of anti-inflammatory drugs in the presence of cyclic stretching also leads to the development of tendinopathy (Li et al., 2004). This suggests that anti-inflammatory drugs are not able to suppress the immune response caused by cyclic stretching. Besides, there are currently no exact control and preventive methods for the inflammatory response resulting from such cyclic stretching or overloading. However, on the positive side, appropriate external forces also play a role in tendon regeneration. In one treatment modality, in order to restore normal ECM structure, tendons have been shown to respond to external tensile loading during healing (Freedman et al., 2018). Recently, dynamic stretching and substrates with mechanical property gradients have been reported to promote the differentiation of stem cells into tenocytes (Liu et al., 2017) and increase matrix production by differentiated tenocytes (Deng et al., 2014). Additionally, a multi-scale computational model for investigating the relationship between loading and tendon healing has been built (Chen K. et al., 2018). This model shows that the magnitude and application time of loading are crucial to tendon healing and regulation of collagen synthesis (Packer et al., 2014). Besides, the immune response is also regulated by stretch similar to biomechanics or mechanical loading provided by biomaterials or mechanical devices in tendon regeneration. In a recent study, mechanical loading appears to regulate M1 phase of inflammation, which improves tendon regeneration (Blomgran et al., 2016). Mechanosensitive proteins or genes contribute to immune responses during the tendon healing process as well. One mechanosensitive transcription factor, known as early growth response-1 (EGR1), plays a key role in the inflammatory response (Soo et al., 2006), and overexpression of EGR1 has been demonstrated to promote tendon healing (Gaut et al., 2016). This indicates the potential value of mechanosensitive protein in the regulation of immune response. As known, the properties of biomaterials such as tension within the fibers can modulate tendon healing capacity (Brammer et al., 2012; Zhang et al., 2017). Furthermore, whether the effects of the immune response on tendon healing can be modulated by biomaterials is an intriguing topic. To investigate that, a study showed that highly aligned fibers mitigate adverse tendon fibroblast response to paracrine signals or secreted pro-inflammatory cytokines of macrophages, by means of changing the matrix topography (Schoenenberger et al., 2018), and in this case, macrophages also showed a trend to M2-like polarization (Schoenenberger et al., 2020). This means that biomimetic scaffolds will regulate tendon resident cells’ response to inflammation and even affect the development of inflammation through the force between scaffold fibers.

The interplay between forces and immune response has been widely observed in animal models and clinical trials. Because forces are more easily controllable, most applications are based on mechanical regulation. For example, biomechanics provided by biomaterial scaffold fibers can regulate inflammatory activation in rat tendons (Schoenenberger et al., 2020). In clinical trials, strength training provides stretch forces that mitigate the detrimental effects of inflammation (Lee et al., 2012; Hoogvliet et al., 2013). The complex relationship between forces and inflammation during the process of tendon healing suggests a delicate balance that modulates tendon functional regeneration. However, the mechanisms by which tendon tissues maintain the fine balance between their mechanosensitive components and the immune response are still largely unknown. Generally speaking, we should analyze the molecular mechanisms and interactions of such interplay and fine balance to realize the future potential of tendon functional regeneration.

## Conclusion

Immune responses in tendon healing have been studied for decades (Sharma and Maffulli, 2005). The participation of immune components is already well-known, but we still lack a systematic and widely accepted understanding of the mechanistic pathway of the tendon healing process and its negative result—tendon diseases. As a result, tendon diseases remain an intractable clinical problem. The imbalanced anabolic–catabolic responses during the development of inflammation results in matrix degradation and disordered structure, which impairs tendon tensile strength. Moreover, the recovery of normal tendon biomechanical properties and matrix is the basis of tendon functional regeneration. However, the contribution of collagen, elastin, and other macromolecular components to tendon biomechanics remains a systematical extrapolation. The limited knowledge and poor understanding of tendon biomechanical properties and matrix leads to limitations on tendon healing. On the other hand, the effects of mechanics is well-recognized at the cellular level, as mentioned above, but it is not deep enough to further improve tendon healing *in vivo* by way of external forces because the suitable range of external forces is still unclear. Even though a large number of studies on its positive effects has been carried out in animal models and clinical trials, there are few papers at the clinical level that explore the mechanisms of moderate loading on tendon healing. Currently, our knowledge of mechanosensitive proteins and genes is still developing. In conclusion, the deficiency of studies on the interplay between tendon immune response and internal/external forces is one of the limiting factors that hinder tendon functional regeneration.

Taken together, it is necessary to consider both the immune response and internal/external forces to achieve a better understanding of the mechanisms by which these contribute to tendon functional regeneration. The level of immune response is one of the key factors that determine the recovery of tendon internal forces. Additionally, mechanical changes caused by the exertion of internal or external forces on the tendon also modulate the immune response during tendon healing. It is hypothesized that tendon functional regeneration may be achieved by tuning up the immune response and internal/external forces.

## Author Contributions

YY, BH, and HL: conception, design, and manuscript writing. YW and XY: conception and design. KZ and DW: collection and assembly of data. JZ: manuscript writing. HO: conception, design, and final approval of manuscript. All authors contributed to the article and approved the submitted version.

## Conflict of Interest

The authors declare that the research was conducted in the absence of any commercial or financial relationships that could be construed as a potential conflict of interest.

## References

[B1] Abraham A. C. Shah S. A. Thomopoulos S. (2017). Targeting inflammation in rotator cuff tendon degeneration and repair. *Tech. Shoulder Elb. Surg.* 18 84–90. 10.1097/BTE.0000000000000124 28947893 PMC5609736

[B2] al-Qattan M. M. Posnick J. C. Lin K. Y. Thorner P. (1993). Fetal tendon healing: development of an experimental model. *Plast. Reconstr. Surg.* 92 1155–1160; discussion 1161. 8234513

[B3] Alexopoulos L. G. Haider M. A. Vail T. P. Guilak F. (2003). Alterations in the mechanical properties of the human chondrocyte pericellular matrix with osteoarthritis. *J. Biomech. Eng.* 125 323–333. 10.1115/1.1579047 12929236

[B4] Alim M. A. Ackermann P. W. Eliasson P. Blomgran P. Kristiansson P. Pejler G. (2017). Increased mast cell degranulation and co-localization of mast cells with the NMDA receptor-1 during healing after Achilles tendon rupture. *Cell Tissue Res.* 370 451–460. 10.1007/s00441-017-2684-y 28975451

[B5] Andarawis-Puri N. Flatow E. L. Soslowsky L. J. (2015). Tendon basic science: development, repair, regeneration, and healing. *J. Orthop. Res.* 33 780–784. 10.1002/jor.22869 25764524 PMC4427041

[B6] Behzad H. Sharma A. Mousavizadeh R. Lu A. Scott A. (2013). Mast cells exert pro-inflammatory effects of relevance to the pathophyisology of tendinopathy. *Arthritis Res. Ther.* 15:R184. 10.1186/ar4374 24517261 PMC3978883

[B7] Best K. T. Lee F. K. Knapp E. Awad H. A. Loiselle A. E. (2019). Deletion of NFKB1 enhances canonical NF-κB signaling and increases macrophage and myofibroblast content during tendon healing. *Sci. Rep.* 9:10926. 10.1038/s41598-019-47461-5 31358843 PMC6662789

[B8] Bi Y. Ehirchiou D. Kilts T. M. Inkson C. A. Embree M. C. Sonoyama W. (2007). Identification of tendon stem/progenitor cells and the role of the extracellular matrix in their niche. *Nat. Med.* 13 1219–1227. 10.1038/nm1630 17828274

[B9] Birk D. E. Southern J. F. Zycband E. I. Fallon J. T. Trelstad R. L. (1989). Collagen fibril bundles: a branching assembly unit in tendon morphogenesis. *Development* 107 437–443. 2612371 10.1242/dev.107.3.437

[B10] Birk D. E. Zycband E. (1994). Assembly of the tendon extracellular matrix during development. *J. Anat.* 184 457–463. 7928635 PMC1259954

[B11] Blomgran P. Blomgran R. Ernerudh J. Aspenberg P. (2016). A possible link between loading, inflammation and healing: immune cell populations during tendon healing in the rat. *Sci. Rep.* 6:29824. 10.1038/srep29824 27405922 PMC4942825

[B12] Blomgran P. Hammerman M. Aspenberg P. (2017). Systemic corticosteroids improve tendon healing when given after the early inflammatory phase. *Sci. Rep.* 7:12468. 10.1038/s41598-017-12657-0 28963482 PMC5622078

[B13] Brammer K. S. Frandsen C. J. Jin S. (2012). TiO2 nanotubes for bone regeneration. *Trends Biotechnol.* 30 315–322. 10.1016/j.tibtech.2012.02.005 22424819

[B14] Brophy R. H. Kovacevic D. Imhauser C. W. Stasiak M. Bedi A. Fox A. J. S. (2011). Effect of short-duration low-magnitude cyclic loading versus immobilization on tendon-bone healing after ACL reconstruction in a rat model. *J. Bone Joint. Surg. Ser. A* 93 381–393. 10.2106/JBJS.I.00933 21325590 PMC3033202

[B15] Carlson G. A. Dragoo J. L. Samimi B. Bruckner D. A. Bernard G. W. Hedrick M. (2004). Bacteriostatic properties of biomatrices against common orthopaedic pathogens. *Biochem. Biophys. Res. Commun.* 321 472–478. 10.1016/j.bbrc.2004.06.165 15358200

[B16] Chamberlain C. S. Clements A. E. B. Kink J. A. Choi U. Baer G. S. Halanski M. A. (2019). Extracellular vesicle-educated macrophages promote early achilles tendon healing. *Stem Cells* 37 652–662. 10.1002/stem.2988 30720911 PMC6850358

[B17] Chen K. Hu X. Blemker S. S. Holmes J. W. (2018). Multiscale computational model of Achilles tendon wound healing: untangling the effects of repair and loading. *PLoS Comput. Biol.* 14:1006652. 10.1371/journal.pcbi.1006652 30550566 PMC6310293

[B18] Chen M. Guo W. Gao S. Hao C. Shen S. Zhang Z. (2018). Biomechanical stimulus based strategies for meniscus tissue engineering and regeneration. *Tissue Eng. Part B Rev.* 24 392–402. 10.1089/ten.teb.2017.0508 29897012

[B19] Chen Q. Zhou J. Zhang B. Chen Z. Luo Q. Song G. (2018). Cyclic stretching exacerbates tendinitis by enhancing NLRP3 inflammasome activity via F-Actin depolymerization. *Inflammation* 41 1731–1743. 10.1007/s10753-018-0816-5 29951874

[B20] Chen X. M. Cui L. G. He P. Shen W. W. Qian Y. J. Wang J. R. (2013). Shear wave elastographic characterization of normal and torn Achilles tendons: a pilot study. *J. Ultrasound Med.* 32 449–455. 10.7863/jum.2013.32.3.449 23443185

[B21] Chisari E. Rehak L. Khan W. S. Maffulli N. (2020). The role of the immune system in tendon healing: a systematic review. *Br. Med. Bull.* 133 49–64. 10.1093/bmb/ldz040 32163543

[B22] Collinsworth A. M. Zhang S. Kraus W. E. Truskey G. A. (2002). Apparent elastic modulus and hysteresis of skeletal muscle cells throughout differentiation. *Am. J. Physiol. Cell Physiol.* 283 1219–1227. 10.1152/ajpcell.00502.2001 12225985

[B23] Dakin S. G. Dudhia J. Smith R. K. W. (2014). Resolving an inflammatory concept: the importance of inflammation and resolution in tendinopathy. *Vet. Immunol. Immunopathol.* 158 121–127. 10.1016/j.vetimm.2014.01.007 24556326 PMC3991845

[B24] Dakin S. G. Newton J. Martinez F. O. Hedley R. Gwilym S. Jones N. (2018). Chronic inflammation is a feature of Achilles tendinopathy and rupture. *Br. J. Sports Med.* 52 359–367. 10.1136/bjsports-2017-098161 29118051 PMC5867427

[B25] De La Durantaye M. Piette A. B. Van Rooijen N. Frenette J. (2014). Macrophage depletion reduces cell proliferation and extracellular matrix accumulation but increases the ultimate tensile strength of injured Achilles tendons. *J. Orthop. Res.* 32 279–285. 10.1002/jor.22504 24307236

[B26] Deng D. Wang W. Wang B. Zhang P. Zhou G. Zhang W. J. (2014). Repair of Achilles tendon defect with autologous ASCs engineered tendon in a rabbit model. *Biomaterials* 35 8801–8809. 10.1016/j.biomaterials.2014.06.058 25069604

[B27] Dirrichs T. Quack V. Gatz M. Tingart M. Kuhl C. K. Schrading S. (2016). Shear Wave elastography (SWE) for the evaluation of patients with tendinopathies. *Acad. Radiol.* 23 1204–1213. 10.1016/j.acra.2016.05.012 27318786

[B28] Docheva D. Müller S. A. Majewski M. Evans C. H. (2015). Biologics for tendon repair. *Adv. Drug Deliv. Rev.* 84 222–239. 10.1016/j.addr.2014.11.015 25446135 PMC4519231

[B29] Ehrlich H. P. Lambert P. A. Saggers G. C. Myers R. L. Hauck R. M. (2005). Dynamic changes appearing in collagen fibers during intrinsic tendon repair. *Ann. Plast. Surg.* 54 201–206. 10.1097/01.sap.0000141380.52782.db 15655474

[B30] Ekstrand J. Krutsch W. Spreco A. van Zoest W. Roberts C. Meyer T. (2020). Time before return to play for the most common injuries in professional football: a 16-year follow-up of the UEFA Elite Club Injury Study. *Br. J. Sports Med.* 54 421–426. 10.1136/bjsports-2019-100666 31182429 PMC7146935

[B31] Evans R. B. Thompson D. E. (1993). The application of force to the healing tendon. *J. Hand Ther. Off. J. Am. Soc. Hand Ther.* 6 266–284. 10.1016/s0894-1130(12)80328-0 8124441

[B32] Fahy N. Menzel U. Alini M. Stoddart M. J. (2019). Shear and dynamic compression modulates the inflammatory phenotype of human monocytes in vitro. *Front. Immunol.* 10:383. 10.3389/fimmu.2019.00383 30891042 PMC6411641

[B33] Feichtinger X. Monforte X. Keibl C. Hercher D. Schanda J. Teuschl A. H. (2019). Substantial biomechanical improvement by extracorporeal shockwave therapy after surgical repair of rodent chronic rotator cuff tears. *Am. J. Sports Med.* 47 2158–2166. 10.1177/0363546519854760 31206305

[B34] Feng Y. N. Li Y. P. Liu C. L. Zhang Z. J. (2018). Assessing the elastic properties of skeletal muscle and tendon using shearwave ultrasound elastography and MyotonPRO. *Sci. Rep.* 8:17064. 10.1038/s41598-018-34719-7 30459432 PMC6244233

[B35] Ferguson M. W. J. O’Kane S. (2004). Scar-free healing: from embryonic mechanism to adult therapeutic intervention. *Philos. Trans. R. Soc. B Biol. Sci.* 359 839–850. 10.1098/rstb.2004.1475 15293811 PMC1693363

[B36] Frankewycz B. Penz A. Weber J. da Silva N. P. Freimoser F. Bell R. (2018). Achilles tendon elastic properties remain decreased in long term after rupture. *Knee Surg. Sports Traumatol. Arthrosc.* 26 2080–2087. 10.1007/s00167-017-4791-4 29147741

[B37] Freedman B. R. Rodriguez A. B. Leiphart R. J. Newton J. B. Ban E. Sarver J. J. (2018). Dynamic loading and tendon healing affect multiscale tendon properties and ECM stress transmission. *Sci. Rep.* 8:10854. 10.1038/s41598-018-29060-y 30022076 PMC6052000

[B38] Gaut L. Duprez D. (2016). Tendon development and diseases. *Wiley Interdiscip. Rev. Dev. Biol.* 5 5–23. 10.1002/wdev.201 26256998

[B39] Gaut L. Robert N. Delalande A. Bonnin M. A. Pichon C. Duprez D. (2016). EGR1 regulates transcription downstream of mechanical signals during tendon formation and healing. *PLoS One* 11:0166237. 10.1371/journal.pone.0166237 27820865 PMC5098749

[B40] Gelberman R. H. Linderman S. W. Jayaram R. Dikina A. D. Sakiyama-Elbert S. Alsberg E. (2017). Combined administration of ASCs and BMP-12 promotes an M2 macrophage phenotype and enhances tendon healing. *Clin. Orthop. Relat. Res.* 475 2318–2331. 10.1007/s11999-017-5369-7 28462460 PMC5539027

[B41] Guilak F. Fermor B. Keefe F. J. Kraus V. B. Olson S. A. Pisetsky D. S. (2008). The role of biomechanics and inflammation in cartilage injury and repair. *Clin. Orthop. Relat. Res.* 23 1–7. 10.1038/jid.2014.371 15232421

[B42] Guney A. Vatansever F. Karaman I. Kafadar I. H. Oner M. Turk C. Y. (2015). Biomechanical properties of achilles tendon in diabetic vs non-diabetic patients. *Exp. Clin. Endocrinol. Diabetes* 123 428–432. 10.1055/s-0035-1549889 25918879

[B43] He Z. Leong D. J. Xu L. Hardin J. A. Majeska R. J. Schaffler M. B. (2019). CITED2 mediates the cross-talk between mechanical loading and IL-4 to promote chondroprotection. *Ann. N. Y. Acad. Sci.* 1442 128–137. 10.1111/nyas.14021 30891766 PMC6956611

[B44] Hill J. R. Eekhoff J. D. Brophy R. H. Lake S. P. (2020). Elastic fibers in orthopedics: form and function in tendons and ligaments, clinical implications, and future directions. *J. Orthop. Res.* 38 2305–2317. 10.1002/jor.24695 32293749 PMC7572591

[B45] Hoogvliet P. Randsdorp M. S. Dingemanse R. Koes B. W. Huisstede B. M. A. (2013). Does effectiveness of exercise therapy and mobilization techniques offer guidance for the treatment of lateral and medial epicondylitis? A systematic review. *Br. J. Sports Med.* 47 1112–1119. 10.1136/bjsports-2012-091990 23709519

[B46] Hope M. Saxby T. S. (2007). Tendon healing. *Foot Ankle Clin.* 12 553–567. 10.1016/j.fcl.2007.07.003 17996614

[B47] Hudgens J. L. Sugg K. B. Grekin J. A. Gumucio J. P. Bedi A. Mendias C. L. (2016). Platelet-Rich plasma activates proinflammatory signaling pathways and induces oxidative stress in tendon fibroblasts. *Am. J. Sports Med.* 44 1931–1940. 10.1177/0363546516637176 27400714 PMC4970921

[B48] Hurle J. M. Corson G. Daniels K. Reiter R. S. Sakai L. Y. Solursh M. (1994). Elastin exhibits a distinctive temporal and spatial pattern of distribution in the developing chick limb in association with the establishment of the cartilaginous skeleton. *J. Cell Sci.* 107 2623–2634. 7844176 10.1242/jcs.107.9.2623

[B49] Ingraham J. M. Hauck R. M. Ehrlich H. P. (2003). Is the tendon embryogenesis process resurrected during tendon healing? *Plast. Reconstr. Surg.* 112 844–854. 10.1097/01.PRS.0000070180.62037.FC 12960868

[B50] Jetten N. Verbruggen S. Gijbels M. J. Post M. J. De Winther M. P. J. Donners M. M. P. C. (2014). Anti-inflammatory M2, but not pro-inflammatory M1 macrophages promote angiogenesis in vivo. *Angiogenesis* 17 109–118. 10.1007/s10456-013-9381-6 24013945

[B51] Kannus P. (2000). Structure of the tendon connective tissue. *Scand. J. Med. Sci. Sport* 10 312–320. 10.1034/j.1600-0838.2000.010006312.x 11085557

[B52] Klatte-Schulz F. Minkwitz S. Schmock A. Bormann N. Kurtoglu A. Tsitsilonis S. (2018). Different achilles tendon pathologies show distinct histological and molecular characteristics. *Int. J. Mol. Sci.* 19:404. 10.3390/ijms19020404 29385715 PMC5855626

[B53] Koob T. J. Summers A. P. (2002). Tendon–bridging the gap. *Comp. Biochem. Physiol. A Mol. Integr. Physiol.* 133 905–909. 10.1016/S1095-6433(02)00255-6 12485682

[B54] Lee B. G. Cho N. S. Rhee Y. G. (2012). Effect of two rehabilitation protocols on range of motion and healing rates after arthroscopic rotator cuff repair: aggressive versus limited early passive exercises. *Arthrosc. J. Arthrosc. Relat. Surg.* 28 34–42. 10.1016/j.arthro.2011.07.012 22014477

[B55] Łęgosz P. Drela K. Pulik Ł Sarzyńska S. Małdyk P. (2018). Challenges of heterotopic ossification—Molecular background and current treatment strategies. *Clin. Exp. Pharmacol. Physiol.* 45 1229–1235. 10.1111/1440-1681.13025 30144316

[B56] Lehner C. Gehwolf R. Ek J. Korntner S. Bauer H. Bauer H.-C. (2016). The blood-tendon barrier: identification and characterisation of a novel tissue barrier in tendon blood vessels. *Eur. Cells Mater.* 31 296–311. 10.22203/ecm.v031a19 27227787

[B57] Lemme N. J. Li N. Y. Kleiner J. E. Tan S. DeFroda S. F. Owens B. D. (2019). Epidemiology and video analysis of achilles tendon ruptures in the national basketball association. *Am. J. Sports Med.* 47 2360–2366. 10.1177/0363546519858609 31268773

[B58] Li H. Y. Hua Y. H. (2016). Achilles tendinopathy: current concepts about the basic science and clinical treatments. *Biomed Res. Int.* 2016:6492597. 10.1155/2016/6492597 27885357 PMC5112330

[B59] Li Z. Yang G. Khan M. Stone D. Woo S. L. Y. Wang J. H. C. (2004). Inflammatory response of human tendon fibroblasts, to cyclic mechanical stretching. *Am. J. Sports Med.* 32 435–440. 10.1177/0095399703258680 14977670

[B60] Lin J. Zhou W. Han S. Bunpetch V. Zhao K. Liu C. (2018). Cell-material interactions in tendon tissue engineering. *Acta Biomater.* 70 1–11. 10.1016/j.actbio.2018.01.012 29355716

[B61] Liu Y. Feng L. Wang H. Wang Y. Chan H.-C. Jiang X. (2018). Identification of an anti-inflammation protein, annexin A1, in tendon derived stem cells (TDSCs) of cystic fibrosis mice: a comparative proteomic analysis. *Proteomics Clin. Appl.* 12:1700162. 10.1002/prca.201700162 29781578

[B62] Liu Y. Xu J. Xu L. Wu T. Sun Y. Lee Y.-W. (2017). Cystic fibrosis transmembrane conductance regulator mediates tenogenic differentiation of tendon-derived stem cells and tendon repair: accelerating tendon injury healing by intervening in its downstream signaling. *FASEB J. Off. Publ. Fed. Am. Soc. Exp. Biol.* 31 3800–3815. 10.1096/fj.201601181R 28495756

[B63] Longo U. G. Ronga M. Maffulli N. (2009). Achilles tendinopathy. *Sports Med. Arthrosc.* 17 112–126. 10.1097/JSA.0000000000000185 19440139

[B64] Maeda E. Ye S. Wang W. Bader D. L. Knight M. M. Lee D. A. (2012). Gap junction permeability between tenocytes within tendon fascicles is suppressed by tensile loading. *Biomech. Model. Mechanobiol.* 11 439–447. 10.1007/s10237-011-0323-1 21706231

[B65] Maffulli N. Longo U. G. Denaro V. (2010). Novel approaches for the management of tendinopathy. *J. Bone Joint Surg. Ser. A* 92 2604–2613. 10.2106/JBJS.I.01744 21048180

[B66] Magne D. Bougault C. (2015). What understanding tendon cell differentiation can teach us about pathological tendon ossification. *Histol. Histopathol.* 30 901–910. 10.14670/HH-11-614 25851144

[B67] Mauro A. Russo V. Di Marcantonio L. Berardinelli P. Martelli A. Muttini A. (2016). M1 and M2 macrophage recruitment during tendon regeneration induced by amniotic epithelial cell allotransplantation in ovine. *Res. Vet. Sci.* 105 92–102. 10.1016/j.rvsc.2016.01.014 27033915

[B68] McBride D. J. Trelstad R. L. Silver F. H. (1988). Structural and mechanical assessment of developing chick tendon. *Int. J. Biol. Macromol.* 10 194–200. 10.1016/0141-8130(88)90048-7

[B69] Menzies N. A. Gomez G. B. Bozzani F. Chatterjee S. Foster N. Baena I. G. (2016). Cost-effectiveness and resource implications of aggressive action on tuberculosis in China, India, and South Africa: a combined analysis of nine models. *Lancet Glob. Heal.* 4 e816–e826. 10.1016/S2214-109X(16)30265-0 PMC5527122 27720689

[B70] Millar N. L. Hueber A. J. Reilly J. H. Xu Y. Fazzi U. G. Murrell G. A. C. (2010). Inflammation is present in early human tendinopathy. *Am. J. Sports Med.* 38 2085–2091. 10.1177/0363546510372613 20595553

[B71] Millar N. L. Murrell G. A. C. Mcinnes I. B. (2017). Inflammatory mechanisms in tendinopathy - towards translation. *Nat. Rev. Rheumatol.* 13 110–122. 10.1038/nrrheum.2016.213 28119539

[B72] Millar N. L. Wei A. Q. Molloy T. J. Bonar F. Murrell G. A. C. (2008). Cytokines and apoptosis in supraspinatus tendinopathy. *Clin. Orthop. Relat. Res.* 466 1569–1576. 10.1007/s11999-008-0265-9 18459030 PMC2505259

[B73] Monti E. Miyagi T. (2015). Structure and function of mammalian sialidases. *Top. Curr. Chem.* 366 185–208. 10.1007/128_2012_328 22760823

[B74] Musson D. S. Naot D. Chhana A. Matthews B. G. McIntosh J. D. Lin S. T. C. (2015). In vitro evaluation of a novel non-mulberry silk scaffold for use in tendon regeneration. *Tissue Eng. Part A* 21 1539–1551. 10.1089/ten.tea.2014.0128 25604072 PMC4426299

[B75] Noah A. C. Li T. M. Martinez L. M. Wada S. Swanson J. B. Disser N. P. (2020). Adaptive and innate immune cell responses in tendons and lymph nodes after tendon injury and repair. *J. Appl. Physiol.* 128 473–482. 10.1152/japplphysiol.00682.2019 31944888 PMC7099435

[B76] Nordez A. Hug F. (2010). Muscle shear elastic modulus measured using supersonic shear imaging is highly related to muscle activity level. *J. Appl. Physiol.* 108 1389–1394. 10.1152/japplphysiol.01323.2009 20167669

[B77] Oshiro W. Lou J. Xing X. Tu Y. Manske P. R. (2003). Flexor tendon healing in the rat: a histologic and gene expression study. *J. Hand Surg. Am.* 28 814–823. 10.1053/S0363-5023(03)00366-6 14507513

[B78] Packer J. D. Bedi A. Fox A. J. Gasinu S. Imhauser C. W. Stasiak M. (2014). Effect of immediate and delayed high-strain loading on tendon-to-bone healing after anterior cruciate ligament reconstruction. *J. Bone Joint Surg. Am. Vol.* 96 770–777. 10.2106/JBJS.L.01354 24806014 PMC4001459

[B79] Pailler-Mattei C. Bec S. Zahouani H. (2008). In vivo measurements of the elastic mechanical properties of human skin by indentation tests. *Med. Eng. Phys.* 30 599–606. 10.1016/j.medengphy.2007.06.011 17869160

[B80] Pang X. Wu J. P. Allison G. T. Xu J. Rubenson J. Zheng M. H. (2017). Three dimensional microstructural network of elastin, collagen, and cells in Achilles tendons. *J. Orthop. Res.* 35 1203–1214. 10.1002/jor.23240 27002477

[B81] Raghunathan V. K. Thomasy S. M. Strøm P. Yañez-Soto B. Garland S. P. Sermeno J. (2017). Tissue and cellular biomechanics during corneal wound injury and repair. *Acta Biomater.* 58 291–301. 10.1016/j.actbio.2017.05.051 28559158 PMC5560898

[B82] Redaelli A. Vesentini S. Soncini M. Vena P. Mantero S. Montevecchi F. M. (2003). Possible role of decorin glycosaminoglycans in fibril to fibril force transfer in relative mature tendons - A computational study from molecular to microstructural level. *J. Biomech.* 36 1555–1569. 10.1016/S0021-9290(03)00133-7 14499303

[B83] Robinson K. A. Sun M. Barnum C. E. Weiss S. N. Huegel J. Shetye S. S. (2017). Decorin and biglycan are necessary for maintaining collagen fibril structure, fiber realignment, and mechanical properties of mature tendons. *Matrix Biol.* 64 81–93. 10.1016/j.matbio.2017.08.004 28882761 PMC5705405

[B84] Sawadkar P. Player D. Bozec L. Mudera V. (2020). The mechanobiology of tendon fibroblasts under static and uniaxial cyclic load in a 3D tissue engineered model mimicking native extracellular matrix. *J. Tissue Eng. Regen. Med.* 14 135–146. 10.1002/term.2975 31622052

[B85] Schiele N. R. von Flotow F. Tochka Z. L. Hockaday L. A. Marturano J. E. Thibodeau J. J. (2015). Actin cytoskeleton contributes to the elastic modulus of embryonic tendon during early development. *J. Orthop. Res.* 33 874–881. 10.1002/jor.22880 25721681 PMC4889338

[B86] Schoenenberger A. D. Foolen J. Moor P. Silvan U. Snedeker J. G. (2018). Substrate fiber alignment mediates tendon cell response to inflammatory signaling. *Acta Biomater.* 71 306–317. 10.1016/j.actbio.2018.03.004 29530822

[B87] Schoenenberger A. D. Tempfer H. Lehner C. Egloff J. Mauracher M. Bird A. (2020). Macromechanics and polycaprolactone fiber organization drive macrophage polarization and regulate inflammatory activation of tendon in vitro and in vivo. *Biomaterials* 249:120034. 10.1016/j.biomaterials.2020.120034 32315865

[B88] Screen H. R. C. Berk D. E. Kadler K. E. Ramirez F. Young M. F. (2015). Tendon functional extracellular matrix. *J. Orthop. Res.* 33 793–799. 10.1002/jor.22818 25640030 PMC4507431

[B89] Sharma P. Maffulli N. (2005). Tendon injury and tendinopathy. *J. Bone Joint Surg.* 87 187–202. 10.2106/JBJS.D.01850 15634833

[B90] Sherlock J. P. Joyce-Shaikh B. Turner S. P. Chao C. C. Sathe M. Grein J. (2012). IL-23 induces spondyloarthropathy by acting on ROR-γt+ CD3+ CD4- CD8- entheseal resident T cells. *Nat. Med.* 18 1069–1076. 10.1038/nm.2817 22772566

[B91] Soo J. C. Min J. K. Homer R. J. Hye R. K. Zhang X. Lee P. J. (2006). Role of early growth response-1 (Egr-1) in interleukin-13-induced inflammation and remodeling. *J. Biol. Chem.* 281 8161–8168. 10.1074/jbc.M506770200 16439363

[B92] Stauber T. Blache U. Snedeker J. G. (2020). Tendon tissue microdamage and the limits of intrinsic repair. *Matrix Biol.* 85–86 68–79. 10.1016/j.matbio.2019.07.008 31325483

[B93] Stolk M. Klatte-Schulz F. Schmock A. Minkwitz S. Wildemann B. Seifert M. (2017). New insights into tenocyte-immune cell interplay in an in vitro model of inflammation. *Sci. Rep.* 7:9801. 10.1038/s41598-017-09875-x 28851983 PMC5575127

[B94] Sunwoo J. Y. Eliasberg C. D. Carballo C. B. Rodeo S. A. (2020). The role of the macrophage in tendinopathy and tendon healing. *J. Orthop. Res.* 38 1666–1675. 10.1002/jor.24667 32190920

[B95] Thorpe C. T. Chaudhry S. Lei I. I. Varone A. Riley G. P. Birch H. L. (2015). Tendon overload results in alterations in cell shape and increased markers of inflammation and matrix degradation. *Scand. J. Med. Sci. Sport* 25 e381–e391. 10.1111/sms.12333 25639911

[B96] Thorpe C. T. Screen H. R. C. (2016). “Tendon structure and composition,” in *Metabolic Influences on Risk for Tendon Disorders*, eds Ackermann P. W. Hart D. A. (Cham: Springer International Publishing), 3–10. 10.1007/978-3-319-33943-6_1

[B97] Vinhas A. Rodrigues M. T. Gonçalves A. I. Reis R. L. Gomes M. E. (2020). Magnetic responsive materials modulate the inflammatory profile of IL-1β conditioned tendon cells. *Acta Biomater.* 117 235–245. 10.1016/j.actbio.2020.09.028 32966921

[B98] Williams I. F. Heaton A. McCullagh K. G. (1980). Cell morphology and collagen types in equine tendon scar. *Res. Vet. Sci.* 28 302–310. 10.1016/s0034-5288(18)32713-9 7414083

[B99] Wilusz R. E. Zauscher S. Guilak F. (2013). Micromechanical mapping of early osteoarthritic changes in the pericellular matrix of human articular cartilage. *Osteoarthr. Cartil.* 21 1895–1903. 10.1016/j.joca.2013.08.026 24025318 PMC3856176

[B100] Wojciak B. Crossan J. F. (1993). The accumulation of inflammatory cells in synovial sheath and epitenon during adhesion formation in healing rat flexor tendons. *Clin. Exp. Immunol.* 93 108–114. 10.1111/j.1365-2249.1993.tb06505.x 8324895 PMC1554749

[B101] Wu J. Yuan H. Li L. Fan K. Qian S. Li B. (2018). Viscoelastic shear lag model to predict the micromechanical behavior of tendon under dynamic tensile loading. *J. Theor. Biol.* 437 202–213. 10.1016/j.jtbi.2017.10.018 29111420

[B102] Wu P. T. Su W. R. Li C. L. Hsieh J. L. Ma C. H. Wu C. L. (2019). Inhibition of cd44 induces apoptosis, inflammation, and matrix metalloproteinase expression in tendinopathy. *J. Biol. Chem.* 294 20177–20184. 10.1074/jbc.RA119.009675 31732563 PMC6937571

[B103] Wu Y.-F. Wang H.-K. Chang H.-W. Sun J. Sun J.-S. Chao Y.-H. (2017). High glucose alters tendon homeostasis through downregulation of the AMPK/Egr1 pathway. *Sci. Rep.* 7:44199. 10.1038/srep44199 28266660 PMC5339827

[B104] Young N. J. Becker D. L. Fleck R. A. Goodship A. E. Patterson-Kane J. C. (2009). Maturational alterations in gap junction expression and associated collagen synthesis in response to tendon function. *Matrix Biol.* 28 311–323. 10.1016/j.matbio.2009.05.002 19481603

[B105] Zhang C. Wang X. Zhang E. Yang L. Yuan H. Tu W. (2017). An epigenetic bioactive composite scaffold with well-aligned nanofibers for functional tendon tissue engineering. *Acta Biomater.* 66 141–156. 10.1016/j.actbio.2017.09.036 28963019

[B106] Zhang K. Asai S. Hast M. W. Liu M. Usami Y. Iwamoto M. (2016). Tendon mineralization is progressive and associated with deterioration of tendon biomechanical properties, and requires BMP-Smad signaling in the mouse Achilles tendon injury model. *Matrix Biol.* 52–54 315–324. 10.1016/j.matbio.2016.01.015 26825318 PMC4875838

